# Supplementary material for: MetaRibo-Seq measures translation in microbiomes
Source: Nat Commun. 2020 Jun 29;11:3268. doi: 10.1038/s41467-020-17081-z (PMC7324362; doi:10.1038/s41467-020-17081-z)
Supplement: Supplementary file 10 — Supplementary Data 7 [file 41467_2020_17081_MOESM10_ESM.zip › File2/Confidence_High_Taxonomy/188198_out.krona.html]

Javascript must be enabled to view this page.

members
magnitude
magnitudeUnassigned
count
unassigned
taxon
rank

188198\_out

2

2
2
superkingdom

phylum
2
1239

1
class
526524

1
order
526525

128827
family
1

genus
1
100883

665941

SRS148511\_contig\_number\_contig-100\_7647.7648
species
1

class
1
186801

186802

SRS049896\_contig\_number\_contig-100\_6436.175218
order
1
